# Golgi Metal Ion Homeostasis in Human Health and Diseases

**DOI:** 10.3390/cells11020289

**Published:** 2022-01-15

**Authors:** Jie Li, Yanzhuang Wang

**Affiliations:** 1 Department of Molecular, Cellular and Developmental Biology, University of Michigan, 1105 North University Avenue, Ann Arbor, MI 48109-1085, USA; jieltian@umich.edu; 2 Department of Neurology, University of Michigan School of Medicine, Ann Arbor, MI 48109-1085, USA

**Keywords:** Golgi, homeostasis, transporter, channel, metal ion, calcium, manganese, zinc, copper

## Abstract

The Golgi apparatus is a membrane organelle located in the center of the protein processing and trafficking pathway. It consists of sub-compartments with distinct biochemical compositions and functions. Main functions of the Golgi, including membrane trafficking, protein glycosylation, and sorting, require a well-maintained stable microenvironment in the sub-compartments of the Golgi, along with metal ion homeostasis. Metal ions, such as Ca^2+^, Mn^2+^, Zn^2^^+^, and Cu^2+^, are important cofactors of many Golgi resident glycosylation enzymes. The homeostasis of metal ions in the secretory pathway, which is required for proper function and stress response of the Golgi, is tightly regulated and maintained by transporters. Mutations in the transporters cause human diseases. Here we provide a review specifically focusing on the transporters that maintain Golgi metal ion homeostasis under physiological conditions and their alterations in diseases.

## 1. Introduction

The Golgi apparatus is an organelle found in most eukaryotic cells. Being part of the endomembrane system in the cytoplasm, it resides at the intersection of the exocytic and endocytic pathways, and works mainly in post-translational modifications and sorting of lipids and proteins. One unique characteristic of the Golgi is the multilayer stack that divides the Golgi membrane system into several sub-compartments known as *cis-*, medial, and *trans-*Golgi, each of which contains a set of glycosylation enzymes that sequentially remove or add various sugar monomers to proteins as they pass through the Golgi. To fulfill its function, the Golgi structure is highly dynamic, while Golgi structure and function are tightly regulated [1]. Similarly, the microenvironment of each sub-compartment is also under strict regulation in response to intracellular environmental changes.

Generally, Golgi homeostasis requires not only the proper positioning and functioning of glycosylation enzymes and trafficking machineries, but also the regulation and maintenance of Golgi lumenal pH, ion, and redox homeostasis, which have been generally reviewed previously [2]. Metal ions in the Golgi lumen, such as Ca^2+^, Mn^2+^, Zn^2+^, and Cu^2+^, are important cofactors of many Golgi-residing enzymes. Being an indispensable part of Golgi homeostasis, the maintenance of metal ion homeostasis involves the cooperation of ATPase pumps, channels, and metal ion binding proteins. Mutations in these regulators impair metal ion homeostasis, disrupt cellular activities, and cause human diseases (summarized in Table 1). Here we provide a more in-depth review specifically on the regulators involved in Golgi Ca^2+^, Mn^2+^, Zn^2+^, and Cu^2+^ ion homeostasis and their functions under physiological and pathological conditions.

**Table 1:** Human diseases related to Golgi metal ion regulators.

**Metal Ion**	Disease	OMIM	Gene	Protein	Clinical Features
Ca^2+^/Mn^2+^	Brody myopathy (BRM) [3]	601003	*ATP2A1*	SERCA1	Early onset of muscle function disorder characterized by muscle cramping and post-exercise stiffening (myopathy).
	Acrokeratosis verruciformis (AKV) [4]	101900	*ATP2A2*	SERCA2	Early onset keratinization disorder affecting the distal extremities.
	Darier disease (DD) [5]	124200	*ATP2A2*	SERCA2	Early onset keratinizating disorder characterized by small papules predominantly in seborrheic areas.
	Hailey-Hailey disease (HHD) [6]	169600	*ATP2C1*	SPCA1	A skin disease causing persistent blisters and suprabasal cell separation (acantholysis) of the epidermis.
	Spinocerebellar ataxia 15 (SCA15) [7]	606658	*ITPR1*	IP3R 1	A neurological condition characterized by progressive gait and limb ataxia.
	Spinocerebellar ataxia 29 (SCA29) [8]	117360	*ITPR1*	IP3R 1	Early onset cerebellar ataxia causing slowly progressive or non-progressive gait and limb ataxia.
	Gillespie syndrome (GLSP) [9]	206700	*ITPR1*	IP3R 1	A congenital neurological disorder characterized by the association of partial bilateral aniridia with non-progressive cerebellar ataxia, and intellectual disability.
	Anhidrosis, isolated, with normal sweat glands (ANHD) [10]	106190	*ITPR2*	IP3R 2	A disorder characterized by absence of perspiration and subsequent heat intolerance with normal morphology and number of sweat glands.
	Malignant hyperthermia 1 (MHS1) [11]	145600	*RYR1*	RyR1	A skeletal muscle disorder and the main causes of death due to anesthesia characterized by any combination of hyperthermia, skeletal muscle rigidity, tachycardia or arrhythmia, respiratory and metabolic acidosis, and rhabdomyolysis.
	Central core disease of muscle (CCD) [12,13]	117000	*RYR1*	RyR1	A mild congenital myopathy characterized by motor developmental delay and signs of mild proximal weakness.
	Multiminicore disease with external ophthalmoplegia (MMDO) [14]	255320	*RYR1*	RyR1	A heterogeneous neuromuscular disorder characterized by neonatal hypotonia, delayed motor development, and generalized muscle weakness and amyotrophy.
	Arrhythmogenic right ventricular dysplasia, familial, 2 (ARVD2) [15]	600996	*RYR2*	RyR2	A congenital heart disease characterized by effort-induced polymorphic ventricular tachycardias due to large areas of fatty-fibrous replacement in the subepicardial layer of the right ventricle.
	Ventricular tachycardia, catecholaminergic polymorphic, 1 (CPVT1) [16]	604772	*RYR2*	RyR2	An arrhythmogenic disorder characterized by physical activity- or stress-induced, polymorphic ventricular tachycardia that may degenerate into deteriorate into ventricular fibrillation.
	Congenital disorders of glycosylation, Type IIk (CDG2K) [17]	614727	*TMEM165*	TMEM165	An autosomal recessive disorder with a variable phenotype, characterized by growth retardation.
Zn^2+^	A novel syndrome with early onset agammaglobulinemia and absent B cells of unknown cause [18]	*N/A*	*SLC39A7*	Zip7	A novel autosomal recessive disease characterized by absent B cells, agammaglobulinemia and early-onset infections.
	Ehlers–Danlos syndrome, Spndylodysplastic Type, 3 (SCD-EDS) [19]	612350	*SLC39A13*	Zip13	Postnatal growth retardation characterized by short stature, hyperelastic skin and hypermobile joints, protuberant eyes with bluish sclerae, atrophy of the thenar muscles, wrinkled palms and tapering fingers.
Cu^2+^	Menkes disease (MNK) [20,21,22]	309400	*ATP7A*	ATP7A	A disorder characterized by generalized copper deficiency, early retardation in growth, peculiar hair, and focal cerebral and cerebellar degeneration due to the dysfunction of several copper-dependent enzymes.
	Occipital horn syndrome (OHS) [23]	304150	*ATP7A*	ATP7A	A rare connective tissue disorder characterized by hyperelastic and bruisable skin, hernias, bladder diverticula, hyperextensible joints, varicosities, and multiple skeletal abnormalities, sometimes accompanied by mild neurologic impairment, and bony abnormalities of the occiput.
	Distal spinal muscular atrophy, X-linked, 3 (DSMAX3) [24]	300489	*ATP7A*	ATP7A	Neuromuscular disorders caused by selective degeneration of motor neurons in the anterior horn of the spinal cord.
	Wilson disease (WD) [25,26]	277900	*ATP7B*	ATP7B	A disorder characterized by dramatic accumulation of intracellular copper with subsequent hepatic and neurologic abnormalities

## 2. Ca^2+^/Mn^2+^ Transporters and Channels

As one of the most ubiquitous second messengers, Ca^2+^ plays a critical role in a variety of intracellular signaling events in the cell. The resting concentration of Ca^2+^ in the cytoplasm is normally maintained at around 100 nM, while the extracellular level is in low millimolar range. To maintain the low cytosolic concentration, most of the Ca^2+^ is stored in the lumen of the endoplasmic reticulum (ER) and the Golgi. Correlative laser scanning confocal fluorescence microscopy (LSCFM) and ion microscopy revealed that the Golgi is capable of storing up to 5% of total cellular Ca^2+^, depending on the cell type, and is more resistant to Ca^2+^ depletion than other cellular organelles [27]. The maintenance of high Ca^2+^ levels in the Golgi lumen is facilitated by Golgi-residing Ca^2+^ ATPases (transporters), Ca^2+^ release channels, and Ca^2+^ binding proteins. This section focuses on these proteins in the maintenance of Ca^2+^ and Mn^2+^ homeostasis in the Golgi as well as their significance in Golgi function.

### 2.1. Ca^2+^/Mn^2+^ Transporters in the Golgi

The early Golgi compartments host many Ca^2+^ pumps that are also highly abundant in the ER, such as Sarcoplasmic/endoplasmic reticulum calcium ATPases (SERCAs), possibly because of the constant content exchange between the Golgi and ER, mediated by direct contact and vesicles [28,29]. On the other hand, the Golgi also contains unique Ca^2+^ transporters not found in the ER, including Secretory pathway Ca^2+^-transporting ATPases (SPCAs, also known as the Calcium-transporting ATPase type 2C members) and TMEM165 [29]. Different from ER Ca^2+^ pumps, they usually show high affinity to Mn^2+^ ion and therefore act as Mn^2+^ transporters as well.

The human genome has three SERCA genes, ATP2A1, ATP2A2, and ATP2A3, which encode P2A ATPase family members SERCA1 (SERCA 1a, 1b), 2 (SERCA 2a, 2b), and 3 (SERCA 3 a-e), respectively. The major structure of SERCA contains a membrane (M) domain consisting of 10 transmembrane helices containing the ion-binding sites, and a large cytosolic domain including the actuator (A), phosphorylation (P), and nucleotide-binding (N) domains. A highly conserved sequence DKTGTLT in the P-domain is found in all P-type ATPase family members and catalyzes the autophosphorylation of the P-domain [30]. The transport of Ca^2+^ from the cytosol into the Golgi lumen by these P-type ATPases is facilitated by the ATP-hydrolysis-dependent conformational change between the two major statuses (E1 and E2) of the SERCA enzymes. The E1 status presents high Ca^2+^-binding affinity and exposure of the two Ca^2+^-binding sites to the cytosol [31]. The binding of Ca^2+^ and ATP causes phosphorylation on a highly conserved aspartate, leading to a conformational change to the E2 status, which shows lower affinity to the Ca^2+^ and reorients toward the lumenal face. Release of Ca^2+^ and ATP hydrolysis are accompanied by the dephosphorylation that leads to the conformational change back to the E1 state, which represents the end of a Ca^2+^ transport cycle [32].

In human cells, two different genes, ATP2C1 and ATP2C2, encode four SPCA1 splice variants and SPCA2, respectively [33,34]. Similar to SERCAs and other P-type ATPases, SPCA isoforms undergoes Ca^2+^ (or Mn^2+^)-binding-induced autophosphorylation of the conserved asparagine (Asp350 in human SPCA1 and Asp351 in SERCA1a) [35]. The reversible SPCA cycle transferring ion across the membrane is also facilitated by the conformational change between the E1 and E2 statuses with different affinity to the ion and orientation [36]. Different from SERCAs that transport two Ca^2+^ ions upon the hydrolysis of one ATP, the SPCA pumps contain only one Ca^2+^-binding site, therefore, transport only one Ca^2+^ ion into the Golgi lumen per ATP. Besides, the maximal turnover rates of the SPCA ATPase are much lower than that of SERCA1a [37]. Another unique characteristic in contrast to most other Ca^2+^-ATPases is that SPCAs display high affinity toward Mn^2+^ and thus also act as Mn^2+^ transporters. Since only one ion-binding site is conserved in SPCAs, it is suggested that only one Ca^2+^ or Mn^2+^ is transported by the energy derived from the hydrolysis of one ATP molecule. Although both SPCA isoforms catalyze Ca^2+^ or Mn^2+^-dependent autophosphorylation, SPCA1 shows a lower Ca^2+^ affinity and slightly lower Mn^2+^ affinity comparing with SPCA2 [35].

It is widely accepted that SERCAs and SPCAs both contribute to the Ca^2+^ uptake into the Golgi since it is only partially inhibited by the SERCA inhibitor thapsigargin [38,39]. Isolated membranes show enrichment of SERCA in the ER and *cis*-Golgi while SPCA1 in the *trans-*Golgi membranes [40]. This conclusion was supported by immunoelectron microscopy (immuno-EM) [41]. Additionally, investigation using Ca^2+^ probes targeted to the *cis-* and *trans-*Golgi showed that the Ca^2+^ accumulation in these compartments is mainly facilitated by SERCA and SPCA1, respectively [39]. As Golgi-specific Ca^2+^ pumps, SPCAs have been shown to be the major Ca^2+^ pumps that facilitate the Ca^2+^ uptake in the *trans*-Golgi network (TGN).

TMEM165 was first identified in congenital disorders of glycosylation (CDG) [17] and localize to the Golgi [42]. Loss of TMEM165 function interrupts Ca^2+^, pH, and Mn^2+^ homeostasis and causes glycosylation abnormalities, which are restored by Mn^2+^ supplementation. Therefore, TMEM165 is proposed to be a Golgi-localized Ca^2+^/Mn^2+^ antiporter involved in glycosylation regulation [43,44]. Interestingly, TMEM165 is relocated to lysosomes and subjected to lysosomal degradation upon high Mn^2+^ exposure in a Rab7 and Rab5 independent manner, indicating a Mn^2+^-dependent TMEM165 quality control mechanism involving direct targeting of the protein to the lysosomal degradation pathway [45]. The Mn^2+^-induced translocation and lysosomal degradation of TMEM165 was later associated to the function of SPCA1, since the phenotype was rescued by a gain-of-function mutation of SPCA1. This suggests a possible functional link between the two proteins in response to the cytosolic Mn^2+^ change, however the underlying mechanism remains unclear [46]. One of the unanswered questions is why TMEM165 is degraded upon high Mn^2+^ exposure. One proposal is that TMEM165 works as a Mn^2+^ importer using the gradient of Golgi Ca^2+^. TMEM165 degradation would increase cytosolic Mn^2+^ and engage the major Mn^2+^ importer SPCA1 for detoxification. Another hypothesis is that TMEM165 imports Ca^2+^ into the Golgi using the Golgi Mn^2+^ gradient. Then TMEM165 degradation would restrain the Mn^2+^ in the Golgi to avoid further toxication. Therefore, TMEM165 is proposed to function in both directions depending on the combined effects of Ca^2+^ and Mn^2+^ [45].

### 2.2. Ca^2+^-Release Channels in the Golgi

The inositol 1,4,5-trisphosphate (IP3) receptors (IP3Rs) and ryanodine receptors (RyRs) are two major types of Ca^2+^-release channels in the Golgi. They both open at low cytosolic Ca^2+^ and close at high cytosolic Ca^2+^ concentrations. IP3R receptors are encoded by 3 genes (*ITPR1, 2 and 3*) in human, and are found in Golgi membranes [28,47]. These proteins are better characterized as ER-resident Ca^2+^ channels that release Ca^2+^ from the ER lumen into the cytosol in response to the stimulation of IP3 [9]. The RyR family members encoded by 3 genes (*RYR1, 2 and 3*) in humans are the other major types of Ca^2+^ channels that mediate the release of Ca^2+^ into the cytoplasm [48]. The IP3Rs were once considered to be the only Golgi Ca^2+^ channels, since IP3 but not RyR agonists significantly induced Ca^2+^ release from Golgi membrane fractions in the presence of thapsigargin [49]. However, other experiments in rat sympathetic neurons and neonatal cardiac myocytes argued that functional RyRs are found in the *trans*-Golgi and probably take part in Golgi Ca^2+^ release [50,51]. Emetine, an alkaloid, induces Ca^2+^ release from *trans*-Golgi without inducing Ca^2+^ release from the ER. It is unlikely that emetine induces Ca^2+^ release via activation of RyR2 or inhibition of SPCA, and the underlying molecular mechanism requires further investigation [52].

### 2.3. Ca^2+^-Binding Proteins in the Golgi Lumen

In addition to the Ca^2+^ transporters and release channels summarized above, Ca^2+^-binding proteins are another factor involved in Ca^2+^ homeostasis in the Golgi lumen. CALNUC, 45 kDa calcium-binding protein (Cab45), p54/NEFA, and calumenin are identified as major Ca^2+^ binding proteins found specifically in the Golgi lumen. CALNUC (also known as Nucleobindin-1), an EF-hand, Ca^2+^-binding peripheral membrane protein closely linked with the lumenal surface of the *cis-*Golgi cisternae, is the most abundant and well-characterized Golgi Ca^2+^ binding protein [53]. Transiently expressed CALNUC co-distributes with IP3R1 in cells, which increases Ca^2+^ storage as well as Ca^2+^ release upon ATP or IP3 stimulation [47]. Overexpression of SPCA upregulates the expression of CALNUC, indicating that CALNUC, together with SERCA and IP3R, is involved in establishment of the agonist-mobilizable Golgi Ca^2+^ store.

Cab45 is the first ubiquitous Golgi Ca^2+^-binding protein identified. It is a soluble Golgi lumen-resident protein that binds Ca^2+^ via its EF-hands [54]. Retention of Cab45 in the TGN is Ca^2+^-dependent, which is disrupted by treatment of cells with a Ca^2+^ ionophore [55], and is regulated by the Golgi resident serine/threonine kinase Fam20C [56]. Once bound to Ca^2+^, Cab45 oligomerizes and is recruited by secretory proteins. Via super resolution microscopy, the Cab45 oligomer-cargo complex is detected to be preferentially colocalized with the TGN Ca^2+^ pump SPCA1. Phosphorylation of Cab45 by Fam20C reduces the size of Cab45 oligomers, abolishes the TGN retention, and enhances the sorting and secretion of Cab45-cargo complex [56]. These results suggest the possibility that a functional sorting complex formed by Cab45, SPCA1, and cargo protein is regulated by Golgi Ca^2+^ [57].

p54/NEFA [58] and calumenin are two other Ca^2+^-binding proteins found in the Golgi lumen. p54/NEFA is a medial Golgi membrane-associated lumenal protein sharing sequence homology with CALNUC [59]. Calumenin is a glycosylated secretory protein distributed throughout the secretory pathway with a low affinity to Ca^2+^ ions [60,61]. It is reported to interact with RyR2 and SERCA in cardiac and skeletal sarcoplasmic reticulum, and therefore suggested to be a regulator of RyR2 and SERCA, which is tightly coupled with Ca^2+^ cycling [62,63,64].

**Figure 1:**
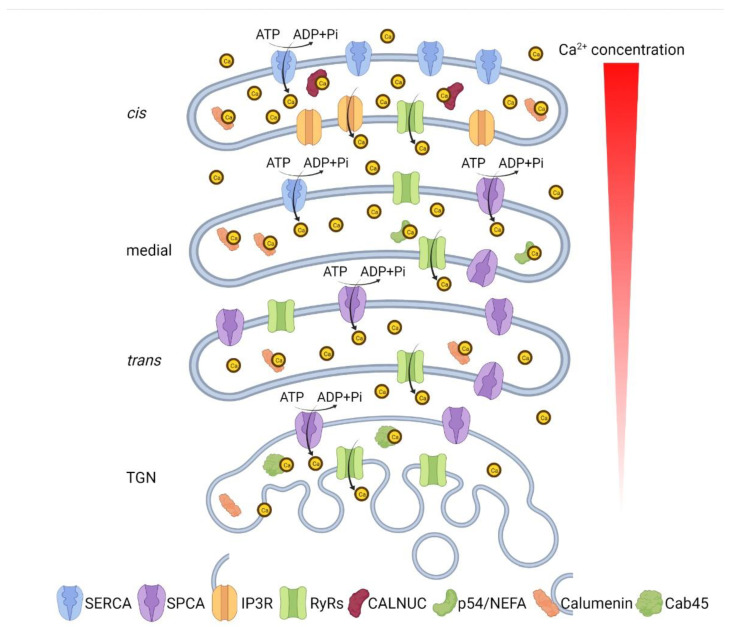
Ca^2+^ concentration and Ca^2+^ homeostasis-related molecules in different Golgi sub-compartments. The *cis*-Golgi expresses mainly SERCA and IP3Rs and contains around 250 µM lumenal Ca^2+^; the medial Golgi mainly expresses SERCA and SPCA1; and the *trans-*Golgi mainly expresses SPCA1 and RyRs, with a lumenal Ca^2+^ of about 130 µM.

In summary, the Golgi is equipped with all the molecular components necessary for maintaining Ca^2+^ homeostasis in the Golgi lumen: Ca^2+^ ATPases that pump Ca^2+^ into the Golgi lumen, Ca^2+^ channels that release Ca^2+^ into the cytoplasm, and Ca^2+^-binding proteins that buffer Ca^2+^ in the Golgi lumen. With all these regulators in action, the distinct sub-compartments of the Golgi appear to have a decreasing lumenal Ca^2+^ concentration through heterogenous expression of Ca^2+^ transporters and channels: the *cis*-Golgi expresses mainly SERCA and IP3Rs and contains around 250 µM lumenal Ca^2+^; the medial Golgi mainly expresses SERCA and SPCA1; and the *trans-*Golgi mainly expresses SPCA1 and RyRs, with a lumenal Ca^2+^ of about 130 µM (Figure 1) [29].

### 2.4. Disruption of Ca^2+^/Mn^2+^ Homeostasis Impairs Golgi Structure and Function

Inhibition of Ca^2+^ pumps was reported to trigger a Golgi morphology change. Knockdown of SPCA1 leads to Golgi ribbon fragmentation, shortened and tubulated cisternae, and complete absence of *cis*- and *trans-*Golgi compartments [41]. In a separate study, transient inhibition of SERCA by thapsigargin-induced Golgi fragmentation before the ER unfolded protein response (UPR) is triggered, indicating an ER-stress-independent Golgi morphology disruption [65]. Further experiments demonstrated that thapsigargin-induced elevation of cytosolic Ca^2+^ activates protein kinase C (PKCα), which subsequently phosphorylates and inactivates GRASP55, a key membrane tether in Golgi stack formation [66,67]. Golgi structural defects also impact membrane trafficking and secretion. Activation of PKCα with phorbol 12-myristate 13-acetate (PMA) and histamine modulates Golgi structure in a similar fashion, indicating a link between Ca^2+^ signaling, Golgi structure and function, and human physiology [37].

Many Golgi-resident glycosylation enzymes such as Golgi α-mannosidases, and proteases such as furin, show Ca^2+^-binding activity [68] or undergoes Ca^2+^-dependent cleavage, which is required for their activation [69]. Taking the Golgi mannosidases as examples, these *cis-*Golgi resident enzymes are required in the early steps of N-glycosylation process, removing mannose residues from a high-mannose intermediate product. Ca^2+^ binds to mannosidases prior to the substrate and remains associated during the enzymatic reaction. Divalent cations, including Mn^2+^, compete with Ca^2+^ and therefore inhibit the mannosidase activity [70].

Mn^2+^ is also a common ion associated with many Golgi-residing glycosylation enzymes, including N-acetylglucosaminyltransferases [71], N-acetylgalactosaminyltransferases [72], mannosyltransferases [73], and some members in the fucosyltransferase family [74,75]. While Mn^2+^ homeostasis in the Golgi lumen is required for proper glycosylation, high cytosolic Mn^2+^ is toxic and related to many human diseases (Table 1) [76,77]. By uptaking Mn^2+^ from cytoplasm into the Golgi lumen, the SPCA pumps avoid cytotoxic accumulation of Mn^2+^, therefore maintain Golgi Mn^2+^ homeostasis and accurate glycosylation [78,79,80]. On the other hand, exposure of Mn^2+^ protects cells from some bacteria-originated toxins (e.g., Shiga toxin, STx) through down-regulation of Golgi phosphoprotein of 130 kDa (GPP130) and protects against STx-induced death [81]. As a type 2 ribosome inactivating protein, STx binds to the cellular toxin receptor to be internalized, and is then transported via the retrograde trafficking route through endosomes and Golgi to the ER, where the toxin is translocated to the cytosol to practice its toxin activity [82,83]. GPP130 serves as a host-cell trafficking receptor that facilitates the intake of the toxin into the TGN via the interaction with Syntaxin 5 [84]. Exposure to Mn^2+^ blocks the retrograde trafficking of STx from endosome to Golgi and leads to its lysosomal degradation [81]. Mn^2+^ targets GPP130 and induces its oligomerization, causing GPP130 redistributed to lysosomes via a clathrin and Rab7-dependent mechanism, where it is subsequently degraded [85,86,87]. Because of its sensitivity to Mn^2+^ exposure, GPP130 can be used as an intra-Golgi Mn^2+^ sensor in Golgi Mn^2+^ homeostasis studies [80].

### 2.5. Golgi Lumenal Ca^2+^ Is Essential for Intra-Golgi Trafficking and Protein Sorting at the TGN

The role of Ca^2+^ in trafficking was first indicated in a study on ER-to-Golgi trafficking in 1989 [88]. Since then, reports on Ca^2+^ participating in intracellular transport events have emerged [89,90,91,92] and Ca^2+^ have been shown to be a fundamental factor in intracellular trafficking [93]. Incubation of isolated Golgi membranes with ionomycin and thapsigargin, which reduce the Golgi lumenal Ca^2+^, inhibits intra-Golgi transport of VSV-G [94]. On the other hand, high Ca^2+^ concentration in the TGN is required for the segregation and sorting of secretory cargo at TGN. This involves the Ca^2+^ pump SPCA1 and Ca^2+^ binding protein Cab45 [55,95,96,97]. SPCA1 binds to the actin-severing protein actin-depolymerizing factor (ADF)/cofilin1 on the TGN via dynamic actin and promotes Ca^2+^ influx into the TGN lumen. Increased Ca^2+^ leads to Cab45 oligomerization and binding to the secretory cargo proteins. The Cab45-cargo complex is then sorted into sphingomyelin-rich vesicles and transported to the plasma membrane for secretion, which is fine-tuned by the Golgi lumenal kinase Fam20C [98]. Knockdown of either SPCA1 or ADF/cofilin1 results in significant mis-sorting of secretory cargo proteins, indicating the role of actin-filaments-induced SPCA1 activation and Ca^2+^ uptake at TGN in protein sorting [96].

### 2.6. Golgi Ca^2+^/Mn^2+^ Homeostasis and Human Diseases

Given the Golgi’s role in protein post-translational modification and sorting, the importance of lumenal Ca^2+^/Mn^2+^ in controlling many of the enzymatic activities within the Golgi, as well as the effect of Ca^2+^/Mn^2+^ fluctuation on the Golgi structure, any changes in Ca^2+^/Mn^2+^ homeostasis are likely to have an impact on Golgi function. Changes in Golgi Ca^2+^/Mn^2+^ handling should, in turn, result in significant changes in cellular activities, contributing to disease pathogenesis [99]. Indeed, mutations in Ca^2+^/Mn^2+^ transporters and channels have been related to various diseases affecting multiple organs and tissues characterized with a wide range of clinical features (summarized in Table 1). In addition, SPCA1 is reported to be required for the maturation and spread of diverse viruses. SPCA1 deficiency leads to impaired virus glycoprotein maturation, reduces the infectivity of furin-requiring viruses, and lowers viral burden in human airway epithelial cells possibly by decreasing the abundance of furin [100]. Importantly, the Spike protein of SARS-CoV-2 virus, which is responsible for the strong infectivity of the virus, contains a furin cleavage site, indicating furin activity as a potential therapeutic target of the wide-spreading COVID-19 [101].

## 3. Zn^2+^ Transporters

Zinc is an indispensable trace element found as a structural or functional component of many proteins [102]. In the cell, Zn^2+^ functions as a structural component, a catalytic factor of many enzymes, and a signaling mediator, and thus participates in many cellular processes [103]. Several enzymes are recognized to respond to the intracellular free Zn^2+^ concentration change, such as protein tyrosine phosphatases [104], phosphodiesterases [105], caspases [106] and kinases [107,108]. The nucleus is the major Zn^2+^ pool in mammalian cells, hosting 30–40% of the cellular Zn^2+^. In the nucleus, Zn^2+^ tightly binds to DNA and RNA synthesis enzymes and transcriptional factors, and influences gene expression [109,110]. The other 50% of Zn^2+^ resides in the cytosol and cytosolic structures, and the remaining 10–20% is stored in membrane-bound organelles [111].

### 3.1. Zn^2+^ Transporters

Zn^2+^ homeostasis in the cell is maintained by two primary types of transporters. Depending on their subcellular location, the Zn^2+^ transporters (ZnTs)/Solute carrier family 30 (SLC30A) member ZnT1-10 transports Zn^2+^ from the cytoplasm into cellular organelles or out of the cell, reducing the cytosolic Zn^2+^, whereas the Zrt- and Irt-like proteins (ZIPs)/ Solute carrier family 39 (SLC39A) member ZIP1-14 transports Zn^2+^ from organelles or external environment into the cytoplasm. Among these, ZnT4, 5, 6, and 7 [112,113,114,115], and ZIP7, 9, 11, and 13 [116,117,118,119], are found in the ER and/or Golgi, and therefore may regulate Zn^2+^ homeostasis in the early secretory pathway. However, the precise localization of some other Zn^2+^ transporters is controversial. For example, overexpressed ZIP13 is localized to the Golgi but endogenous ZIP13 is not, although loss of ZIP13 decreases Zn^2+^ in the secretory pathway [120].

The most established function of ER and/or Golgi Zn^2+^ transporters is to maintain Zn^2+^ homeostasis and supply Zn^2+^ to ectoenzymes in the early secretory pathway [121]. ZnT5 recruits ZnT6 to the ER and Golgi membranes to form ZnT5-ZnT6 heterodimers, which cooperates with ZnT7 homodimers to activate Zn^2+^-associated ectoenzymes, including alkaline phosphatases (ALPs) in the Golgi lumen [122]. In ZnT5-7 triple depleted cells, ALP is degraded by lysosomal and proteasomal pathways, possibly caused by protein misfolding [122]. ALP activity depends on the Zn^2+^ level in the ER and/or Golgi and reflects Zn^2+^ concentration in the early secretory pathway [123]. Therefore, ALP activity is commonly used as a Zn^2+^ indicator in Zn^2+^ homeostasis and transporter-related studies.

### 3.2. Zn^2+^ Function in the Earlier Secretory Pathway

In the Golgi, Zn^2+^ is a cofactor of glycosylation enzymes including α-mannosidase 2 (MAN2A1 and MAN2A2), involved in both the substrate specificity and catalytic activity of the enzyme [124]. In mammary epithelial cells, ZnT4 is identified on the *trans-*Golgi, providing Zn^2+^ for the Zn^2+^-dependent enzyme carbonic anhydrase VI, and regulating its cellular abundance [114]. On the other hand, the activity of β-4-galactosyltransferase, a *trans-*Golgi resident glycosylation enzyme that catalyzes the transfer of galactosyl to N-acetylglucosamine on glycoproteins, is also regulated by the Zn^2+^ level. Excessive Zn^2+^ competes with Mn^2+^, the cofactor of galactosyltransferase, and inhibits the enzyme [125]. Indeed, overexpression of ZnT4 significantly decreased galactosyltransferase activity without affecting the protein abundance. Different from ZnT5 and ZnT6, ZnT4 is localized on *trans-*Golgi instead of *cis-*Golgi, which may explain why it shows no effect on ALP activity [114]. On the other hand, ZIP7, ZIP9, and ZIP13 are reported to export Zn^2+^ from the ER and Golgi lumen to the cytosol, which is also considered to contribute to the function of the early secretory pathway. Endogenous ZIP7 is localized to the Golgi and exports Zn^2+^ from the Golgi lumen [117]. But this conclusion was challenged by another study concluding that only ZIP13 is the Zn^2+^ transporter in the Golgi, while ZIP7 is located in the ER and involved in ER stress regulation [126]. ZIP9 is localized to the *trans-*Golgi independent of Zn^2+^ status; stable expression of ZIP9 reduces ALP activity, indicating its function in Zn^2+^ homeostasis in the secretory pathway [118].

### 3.3. Zn^2+^ Homeostasis and Human Diseases

The release of Zn^2+^ from intracellular stores into the cytosol initiates early or late signaling pathways. The former is transcription-independent, in which Zn^2+^ binds to target receptors; and the latter requires the transcription of Zn^2+^ transport proteins that restore homeostasis [127]. Disruption of Zn^2+^ homeostasis, either by Zn^2+^ deficiency or overload, induces ER stress and UPR, leading to the activation of transcription factors, such as ATF4, ATF6 and XBP1, and upregulation of ZnT and ZIP expression [128,129]. Thus, Zn^2+^ homeostasis is maintained by transcriptional regulation of Zn^2+^ transporters via the ER function.

Disturbance of zinc homeostasis can result in diseases, including cancer [130], diabetes [131,132], neuronal degeneration [133], degenerative disorders of aging [134], and infection and inflammation [135,136]. A number of diseases have been identified to be caused by mutations in Golgi Zn^2+^ transporter genes [18,19] (summarized in Table 1). Some Zn^2+^ transporters are abnormally expressed in Alzheimer’s disease (AD) [137,138], sporadic amyotrophic lateral sclerosis [139], and multiple types of cancer [140,141,142]. These observations reveal a hidden link between abnormal Zn^2+^ accumulation in the Golgi lumen or cytoplasm and pathogenesis, indicating Zn^2+^ transporters as potential therapeutic targets for disease treatment [143,144,145,146].

## 4. Cu Ion (Cu^2+^ and Cu^+^) Transporters

As a cofactor and regulator of many enzymes, copper is required for a variety of vital biological activities, yet toxic as well due to the reactive oxygen species (ROS) produced during its oxidation. Thus, maintenance of cellular Cu homeostasis is critical for cell function and survival [147].

### 4.1. Cu Ion Transporters in the Golgi

Once transported from the blood circulation into the cell by the membrane-localized high affinity copper uptake protein 1 (hCtr1)/SLC31A1, the Cu ion associates with the copper transport protein ATOX1, which transfers it to copper-transporting ATPase-1 (ATP7A) and -2 (ATP7B) on the Golgi membrane. Both ATP7A and B are 8-transmembrane domain containing proteins with six metal-binding domains (MBD) on the cytoplasmic N-terminus. They also contain ATP-binding, phosphatase and phosphorylation domains that regulate their catalytic activity. These ATP-driven pumps acquire Cu ion from the copper chaperones on the cytoplasmic side of the TGN membrane and delivers it into the Golgi lumen to activate Cu-dependent enzymes. Similar to Ca^2+^ ATPases, the Cu transporters belong to the P-type ATPase family and a complete Cu transport cycle involves ATP hydrolysis, catalytic phosphorylation/dephosphorylation, and consequential conformation change that facilitates the transmembrane transport of Cu ions. The import of Cu into Golgi lumen facilitates the incorporation of Cu into Cu-dependent enzymes in the secretory pathway; on the other hand, this avoids the potential toxic accumulation of Cu in the cytoplasm [148,149].

### 4.2. Cu Transporter Trafficking

One unique character of these Cu transporters is their translocation from the TGN to post-Golgi vesicular compartments and subsequently the plasma membrane to export excessive Cu out of the cell in response to a rising Cu level [147,150]. This Cu-induced trafficking is reversible, as the Cu transporters are retrieved back to the TGN once the Cu level is reduced.

Studies have identified some elements responsible for TGN-targeting and Cu-induced trafficking of ATP7A and B, yet detailed mechanisms underlying the trafficking of Cu transporters remain unknown. Deletion of transmembrane domains 3 and 4 results in mis-localization of ATP7A. Furthermore, a 38 amino acid sequence containing transmembrane domain 3 of ATP7A was found to be sufficient for Golgi localization [151]. Disease-related mutants of ATP7A and B are misfolded and degraded by ER-associated degradation (ERAD) [152]. Among the six N-terminal MBDs, MBD 5 or 6 is necessary and sufficient for the copper-induced redistribution, while the first 4 MBDs are not essential [153]. The di-leucine and tri-leucine motifs within the cytosolic C-terminal of ATP7A and B are required for their retrieval to the Golgi [154,155].

Phosphorylation is another factor that affects the trafficking of Cu transporters. Mutation of the phosphorylation sites prevents Cu-induced translocation of ATP7A, while mutations in the phosphatase domain result in hyperphosphorylation of the protein and cause constitutive dislocation from Golgi to the plasma membrane. These findings suggest that Cu-induced trafficking of the Cu-ATPases is associated with the phosphorylated intermediate conformations during catalysis [156,157]. In addition, multiple trafficking factors, including clathrin, AP-1, AP-2, and Rab22, but not caveolin or flotillin, are required for ATP7A trafficking [158].

### 4.3. Cu Homeostasis and Human Diseases

Mutations in Cu transporters have been related to many diseases, including the well-established Menkes disease (MD) and Wilson disease (WD) (summarized in Table 1). An analysis of 36 ATP7A missense mutations identified in MD patients with different phenotypes reveals a correlation between the Cu transport cycle-related phosphorylation/dephosphorylation with the cellular localization of the proteins. Total of 21 loss-of-function mutations have been reported to inhibit catalytic phosphorylation, leading to the retention of ATP7A in the Golgi, while 4 mutants with reduced catalytic dephosphorylation show diffuse post-TGN localization, supporting that phosphorylation and dephosphorylation (possibly conformation change related to the phosphorylation status) are critical for the translocation of ATP7A [159]. In addition, the Cu transport cycle has been implicated in Alzheimer’s and Parkinson’s diseases, drug-resistance in cancer, cancer cell migration, and angiogenesis [160,161,162,163,164], which suggests the Cu transporters as potential beneficial pharmacological targets.

## 5. Summary and Perspective

Herein, we have sought to depict the current understanding of the metal ion homeostasis regulators in the Golgi, emphasizing their functional involvement in physiological conditions. Some questions related to the pathological mechanism of metal ion-related diseases, direct measurement of ion homeostasis in diverse Golgi sub-compartments, Cu trafficking machineries, etc., remain unanswered. Given the Golgi’s role in protein post-translational modification and sorting, as well as the importance of lumenal metal ions in controlling many enzymatic activities, alterations in metal ion homeostasis are likely to affect Golgi function, cause major changes in cellular activities, and contribute to disease pathogenesis. Development of future innovative therapeutic approaches aimed at counteracting the many pathological states associated with Golgi metal ion homeostasis is still challenging.
